# Book Reviews

**Published:** 1898-06

**Authors:** 


A System of Practical Medicine. By American authors. Edited by Alfred Lee Loomis, M. D., late Professor of Pathology and Practical Medicine in the New York University, and William Gilman Thompson, M. D., Professor of Medicine in the New York University. Volume III., pp. 926. Diseases of the alimentary canal, peritoneum, liver and gall bladder, spleen, pancreas and thyroid gland, chronic metal poisoning, alcoholism, morphinism, infectious diseases common to man and animals, miscellaneous subjects. Philadelphia and New York: Lea Brothers & Co. 1898.

In the continuation of this comprehensive system of medicine the third volume presents to our consideration the diseases of the digestive tract. First comes diseases of the alimentary canal, in the consideration of which we find an interesting section devoted to affections of the esophagus, presented by Dr. Allen A. Jones, of Buffalo. This organ is subject to so many changes—traumatic, organic and neurotic,—that it is somewhat surprising that its literature is sparse. Jones has conferred a favor on the profession by grouping within the space of 25 or 30 pages nearly or quite all that is known in regard to esophageal maladies. Foreign bodies and other traumatisms, such as are produced by swallowing corrosive poisons, have been avoided in this monograph, yet we are of the opinion that it would be wise to group in a single brochure the whole subject of diseases and injuries of the esophagus.

The consideration of diseases of the stomach is presented in about 100 pages of interesting text by Dr. Charles G. Stockton and Dr. Allen A. Jones in joint authorship. We have called attention in reviews recently written of treatises on the stomach, lately published, to the importance of a knowledge of these affections by the every-day practitioner of medicine. The routine prescribing of pepsin and bismuth, or other so-called digestive agents, followed by iron as a tonic, one or all, most generally given empirically, forms approximately the be-all and end-all of the average management of digestive disorders. Stockton was one of the first to plead for more accurate diagnosis and better therapeutics in this direction; and he and his associate, Dr. Jones, have done much to develop improvement in this department of internal medicine. We commend what they have here written to careful study by every general practitioner of medicine.

Of scarcely less interest than the foregoing are Diseases of the intestines, which form the subject of two sections written by William W. Johnston and Henry M. Lyman, respectively. Appendicitis is the subject of a section written by W. F. McNutt. Diseases of the peritoneum are presented by Hobart Amory Hare. Since our knowledge becomes more and more perfected with reference to diseases of the appendix, thanks to Mynter, McBurney, Price and others, the field for the peritoneum has become more limited. The "peritonitis" and "inflammation of the bowels" of former days has now easily become appendicitis. Hare, however, discourses briefly and well upon what little is left for the peritoneum in the light of our present pathology.

Coming now to diseases of the large secretive glands we find those of the liver, gall-bladder and ducts written by J. E. Graham; diseases of the spleen by George Roe Lockwood and of the pancreas by Charles G. Stockton. We have lately come to better understand the functions and disorders of this latter organ, the principal functions of which are to convert starch and sugar into glucose, emulsify fats and coagulate casein. Its diseases are not easy to treat and the one most likely to occur—namely, cancer, is incurable. Stockton has brought our knowledge of the subject down to present date in this carefully prepared monograph. Of late years diseases of the thyroid gland are attracting considerable attention, especially cretinism and myxedema. The affections of that organ are well considered by Francis P. Kinnicutt and M. Allen Starr. The remaining sections of the book are devoted to chronic metal poisoning, alcoholism, morphinism, the infectious diseases common to man and animals and a few miscellaneous subjects, such as purpura, hemophilia, diabetes, insolation and the like.

This is a well-arranged and carefully-prepared volume, handsomely printed and bound, and is a credit to the progressiveness and scientific attainments of American physicians.

---

Annual and Analytical Cyclopedia of Practical Medicine. By Charles E. de M. Sajous, M. D., and 100 associate editors, assisted by corresponding editors, collaborators and correspondents. Illustrated with chromo-lithographs, engravings and maps. Volume I. Philadelphia, New York, Chicago: The F. A. Davis Company. 1898.

The issue of the Annual in this new form will be welcomed by the medical profession as an improvement over that which has prevailed during the last ten years. This first volume embraces subjects from abdominal injuries to and including Bright's disease. The first article contains 150 excerpts, besides the general text. This will give an idea as to the exhaustive nature of the references. The abstracts interpolated in the text are controversially arranged, and aimed to sustain the views advanced by authors, or indicate fields as yet insufficiently explored. The work when completed, according to the editor's statement, will present all the general diseases described in text-books on practical subjects, such as medicine, surgery, therapeutics and obstetrics.

It is expected under the general arrangement adopted that the entire field will be completed in six volumes. At all events, the first volume represents one-sixth of the whole number. The book contains numerous illustrations, it is printed in double-columned pages with new clear-faced type, and is handsomely bound. To say that this is a great improvement over the original Sajous' Annual is giving it faint praise in one sense; but in another it speaks loudly in its favor, because the latter was really one of the most useful compilations of medical literature that had ever been printed. This plan, however, surpasses the other in every respect and will be welcomed in the libraries of every practitioner, teacher or writer who has occasion to consult the literature of medicine.

---

An American Text-Book of Genito-Urinary Diseases, Syphilis and Diseases of the Skin. Edited by L. Botton Bangs, M. D., Consulting Surgeon to St. Luke's Hospital and the City Hospital, New York, and to the Methodist Episcopal Hospital, Brooklyn; Visiting Genito-urinary Surgeon to St. Mark's Hospital, New York; Late Professor of Genito-urinary and Venereal Diseases, New York Post Graduate Medical School and Hospital; and W. A. Hardaway, A. M., M. D., Professor of Diseases of the Skin and Syphilis in the Missouri Medical College, St. Louis; Physician for Diseases of the Skin to the Martha Parson's Hospital for Children and to St. John's Hospital, St. Louis. Illustrated with 300 engravings and 20 full-page colored plates. Philadelphia: W. B. Saunders, 925 Walnut street. 1898.

This work, which is designated An American Text-book of Genito-urinary Diseases, Syphilis and Diseases of the Skin, is, as the editors inform us, a collaboration of well-known authorities who have not been restricted in regard to the particular views set forth in its pages, but have been offered every facility for the free expression of individual opinion. It certainly sets forth the clinical facts and the principles of treatment, based on the results of modern research, serving not merely as a text-book for the student and practitioner, but also as a work of reference for the expert. It is essentially clinical and practical in its scope. The first subject treated in the work is on Urine analysis and a consideration of the urine in surgical diseases of the urinary tract, by Paul Thorndike, M. D., in which he describes the characteristics and chemical constituents of urine and the commoner and more practical methods for investigating them in a clear and concise manner, in which the various points are treated at a length proportionate to their intrinsic importance.

International Clinics. A Quarterly of Clinical Lectures and Specially Prepared Articles on Treatment and Drugs. By professors and lecturers in the leading medical colleges of the United States, Germany, Austria, France, Great Britain and Canada. Edited by Judson Daland, M. D. (Univ. of Penna.), Philadelphia, Instructor in Clinical Medicine and Lecturer on Physical Diagnosis in the University of Pennsylvania; Assistant Physician to the Hospital of the University of Pennsylvania, etc.; Fellow of the College of Physicians of Philadelphia. J. Mitchell Bruce, M. D., F. R. C. P., London, England, Physician to, and Lecturer on the Principles and Practice of Medicine at the Charing Cross Hospital. David W. Finlay, M. D., F. R. C. P., Aberdeen, Scotland, Professor of Practice of Medicine in the University of Aberdeen; Physician to, and Lecturer on Clinical Medicine in the Aberdeen Royal Infirmary, etc. Volume I. Eighth series. 1898. Octavo, pp. xii.—363. Philadelphia: J. B. Lippincott Co. 1898.

The lectures in this volume are classified under the following heads: Drugs and remedial agents, treatment, medicine, neurology, surgery, gynecology and obstetrics, ophthalmology, laryngology, rhinology and dermatology. Dr. Charles G. Stockton, of Buffalo, supplies a lecture on Weak heart, and gastrectasis from pyloric spasm. Other prominent lecturers contributing to this volume are Prof. Jaccoud, Byrom Bramwell, E. Fletcher Ingals, Joseph M. Mathews, Charles G. Cumston, A. Pinard, Paul F. Mundé, Henry C. Coe, William Oliver Moore and Samuel G. Dabney. It contains a few illustrations and is excellently printed on lines similar to its predecessors.

Transactions of the Medical Society of the State of North Carolina. Forty-fourth annual meeting, held at Morehead City, N. C., June 8, 9 and 10, 1897. R. D. Jewett, M. D., Secretary, Wilmington, N. C.

This sterling society has again sent out an interesting volume of transactions. Besides the address of the president it contains the annual oration by Dr. Charles O'Hagan Laughinghouse, of Greenville, in which he gave a few hints in medico-social ethics. Then follow the reports of the committees and the papers read at the meeting, including the annual essay by Dr. J. B. Powers, of Wake Forest, entitled Psycho-therapeutics. An obituary of Dr. James McIntosh Hays is accompanied with an excellent half-tone engraving of this distinguished physician whose death was noticed in the JOURNAL for June, 1897. The volume is an excellent record of an interesting meeting.

---

ATLAS AND ESSENTIALS OF PATHOLOGICAL ANATOMY. By PROFESSOR O. BOLLINGER, M. D. Volumes I. and II. Duodecimos, pp. viii.—246 and pp. x.—232. Illustrated. New York: William Wood & Co. 1898.

The study of pathologic anatomy can only be properly pursued in the laboratory, yet aids to its pursuit are necessary in the way of text-books. Illustrations of morbid processes are never very clear unless given in colors. The plates in this atlas are all made from actual specimens and the drawings are true to nature. The colors are in beautiful tints, and taken altogether it is one of the most helpful auxiliaries to the acquirement of definite knowledge in pathologic science. Every laboratory teacher and student should possess it.

LEXIQUE-FORMULAIRE DES NOUVEAUTÉS MÉDICALES. Par LE PROFESSEUR PAUL LEFERT. One vol. in 18–18 de 336 pages, cartonné, 3 fr. Librairie J. B. Baillière et fils, 19 rue Hautefeuille (près du boulevard Saint-Germain), à Paris.

This is a convenient little volume which combines the dictionary and the index of treatment. It is, of course, alphabetically arranged; the terms used are defined; their therapeutic uses given, and in many instances detailed formulæ of their combinations are set forth. It is arranged by a practical physician and is a convenient book of reference for the every-day practitioner of medicine.

---

AIDS TO ASEPTIC TECHNIQUE. By A. D. WHITING, M. D., Assistant Surgeon to the German Hospital, Philadelphia. Duodecimo, pp. 157. Philadelphia: J. B. Lippincott Co. 1898.

The object of this book being to aid those whose duty it is to prepare materials for operations it is addressed more especially to nurses and assistants at the operating table. Nevertheless, it affords pleasant reading to the more experienced practitioner of medicine as well as surgery. It does not aim to take the place of the completer treatise, but rather to reinforce the ready reference library. Every nurse should obtain it and consult it frequently.

---

SEXUAL NEURASTHENIA: Its Hygiene, Causes, Symptoms and Treatment, with a chapter on Diet for the Nervous. By GEORGE M. BEARD, A. M., M. D., formerly Lecturer on Nervous Diseases in the University of the City of New York; Fellow of the New York Academy of Medicine, etc., etc. Edited, with notes and additions, by A. D. Rockwell, A. M., M. D., formerly Professor of Electro-therapeutics in the New York Post-Graduate Medical School and Hospital, etc., etc. With formulas. Fifth edition. Octavo, pp. xii.—308. New York: E. B. Treat & Co. 1898.

To again notice this interesting work seems almost a species of supererogation. The previous four editions have been quickly exhausted and this, the fifth, has been revised with omissions, modifications and additions, so that it is practically an expression of the author's views as at present entertained on all the essential features of which it treats. It occupies a field in which Rockwell stands apparently unrivaled and in which he is accepted as unquestioned authority. He has successfully wrested the treatment of these disorders from the hands of empirics, for which he is entitled to receive the support and thanks of the profession of medicine.

---

PROCEEDINGS OF THE SEVENTH ANNUAL MEETING OF THE ASSOCIATION OF MILITARY SURGEONS OF THE UNITED STATES, held at Columbus, Ohio, May 25, 26 and 27, 1897. Edited by JAMES E. PILCHER, M. D., Captain in the Medical Department of the United States Army; secretary and editor of the Association. Columbus, Ohio: Berlin Printing Co. 1897.

This handsome book is full of interesting material, the importance of which is greatly accentuated now that war with Spain is in actual operation. This association, through its meetings, scientific papers and discussions, has been the means of greatly improving the medical corps of the National guard in the several states. Brought as they have been through its influence into intimate association with regular officers of the army and navy of the United States, much has been learned of the medical and surgical care of troops in the field which will now become available should our forces invade Cuba, as seems probable.

This volume is well edited, beautifully printed and handsomely bound. It should form a part of the library of every military and naval surgeon.

---

TRANSACTIONS OF THE TEXAS STATE MEDICAL ASSOCIATION. Twenty-ninth Annual Session, held at Paris, Texas, April 27, 28, 29 and 30, 1897. H. A. WEST, M. D., Secretary, Galveston, Texas.

The transactions of the Texas state medical association, as published year by year, become an important part of the literature of medicine. The volume before us is no exception to the rule and it testifies to the most excellent condition of the medical profession in the Lone Star State. The book is ably edited by Dr. West, whose labors for the improvement of professional standards are well known throughout the country.

